# The acute respiratory distress syndrome in 2013

**DOI:** 10.1186/2213-0802-1-10

**Published:** 2013-05-17

**Authors:** Michael A Matthay, Yuanlin Song, Chunxue Bai, Kirk D Jones

**Affiliations:** 1 Departments of Medicine, Anesthesia, and Pathology and the, Cardiovascular Research Institute, University of California, San Francisco, USA; 2 Cardiovascular Research Institute, University of California San Francisco, 505 Parnassus Ave, M-917, Box 0624, San Francisco, CA 94143-0624 USA; 3 Department of Pulmonary Medicine, Zhongshan Hospital, Fudan University, Shanghai, 200032 P.R. China

**Keywords:** Lung Injury, Acute Lung Injury, Positive Pressure Ventilation, Oxygenation Index, Cigarette Smoke Exposure

## Abstract

Acute lung injury and the acute respiratory distress syndrome are major causes of morbidity and mortality in critically ill patients. This review focuses on new developments in definitions, epidemiology, clinical and basic research, and promising new directions in treatment. There is new information about the potential contribution of environmental factors, especially exposure to cigarette smoke. Pathologic findings in ARDS have been limited to case reports of open lung biopsies and post-mortem studies but there is some new information from a recent pathology study relative to the frequency of diffuse alveolar damage and the severity of arterial hypoxemia. Further, therapy with lung-protective ventilation and fluid conservative protocol has improved outcomes, but several new trials are in progress to test several promising strategies.

**Electronic supplementary material:**

The online version of this article (doi:10.1186/2213-0802-1-10) contains supplementary material, which is available to authorized users.

There have been several reviews of acute lung injury and the acute respiratory distress syndrome (ALI/ARDS) [1–4]. Most of these reviews have covered the clinical presentation, pathophysiology, pathogenesis, and new treatment options. The primary purpose of this review article is to focus on areas that represent new directions in definitions, epidemiology, clinical and basic research, and the potential for new treatments. In terms of therapy, it is clear that lung-protective ventilation and a fluid conservative strategy have resulted in a major decrease in mortality and a decrease in morbidity in patients with ALI/ARDS [1]. The challenge for the future will be to identify new therapies that can further decrease mortality and reduce short- and long-term morbidity.

## Definitions and epidemiology

Recent studies have demonstrated that it is possible to identify acute lung injury in patients prior to the need for positive pressure ventilation, either by non-invasive ventilation or standard positive pressure ventilation via an endotracheal tube. One study in 2009 published by Levitt et al. established that *early acute lung injury* (EALI) could be identified in the emergency department in patients who presented with bilateral pulmonary infiltrates on the chest radiograph and required more than two liters of nasal oxygen to maintain adequate oxygen saturation in the absence of clinical evidence for left atrial hypertension or chronic lung disease [5]. There were 100 patients identified in this study, and 33 developed acute lung injury (ALI) that required positive pressure ventilation. Subsequently, Gagic and colleagues [6] prospectively derived the Lung Injury Prediction Score (LIPS) in a multi-center study of more than 5,000 patients who were admitted with at least one known risk factor for ALI. In their study, a LIPS score greater than 4 provided the best discrimination with an associated sensitivity of 69%, specificity of 78%, and a positive predictive value of 18%. For some pulmonary-specific risk factors that are included in the LIPS, specifically pneumonia, aspiration, oxygen saturation of less than 95% or a FiO_2_ greater than 35%, the distinction between prevention and early identification is not clear and in fact the patient may already have an element of early lung injury. In other clinical conditions (patients with non-pulmonary sepsis, high-risk elective surgery), there probably is a real distinction in which patients can be identified before they have lung injury. In the Levitt study, the prevalence of ALI was 33% with an approximate median time of progression of less than 24 hours. In the LIPS study, the prevalence of ALI was 7% and progression to ALI developed over a median of two days. Thus, the LIPS criteria have a longer time window to identify patients, but a prevention strategy would need to include more patients, and many of these patients will not develop ALI. The criteria in the Levitt study identified patients with a higher likelihood of progressing to develop ALI requiring positive pressure ventilation, but the time interval for intervention was shorter, so the practical aspects of obtaining consent and enrolling patients in a trial would be more challenging. In one prospective study of more than 300 pediatric patients who developed ALI, Flori et al. [7] identified 20% of children with ALI by oxygenation criteria and chest radiographs, approximately 50% of whom progressed to develop positive pressure-dependent lung injury and the remaining half who did not progress.

Thus, it may well be possible to identify a significant number of patients at an earlier phase of ALI than has been traditionally accomplished in clinical trials. The National Heart Lung and Blood Institute will issue a Request for Proposals (RFP) to focus on the next NHLBI ARDS Network on identifying patients for prevention or early treatment strategies *before* they require positive pressure ventilation. There will need to be experience to establish success of this approach, particularly with the challenges inherent in identifying and enrolling patients in clinical trials over five years.

Recently, an American-European conference and workshop revisited the definitions of acute lung injury and ARDS, and specifically re-evaluated the American-European consensus conference definition from 1994. The result of this workshop has been labeled the Berlin Definition of ARDS [8]. The authors recommended that patients be categorized into three different classifications according to their PaO_2_/FiO_2_ ratios: (A) PaO_2_/FiO_2_ < 300 but >200 mm Hg; (B) PaO_2_/FiO_2_ < 200 but >100 mm Hg; (C) PaO_2_/FiO_2_ < 100 mm Hg. As would be expected, mortality progressively declined in each of these groups. Using a receiver-operating curve, this revised definition showed a small, but significant improvement in the area under the curve from 0.53 to 0.57 compared to the traditional AECC definition, although the absolute difference is small.

Some investigators have carried out clinical trials with modified definitions for the severity of ALI/ARDS according to the anticipated effect of the therapy. For example, Papazian et al. used a PaO_2_/FiO_2_ less than 150 mm Hg to identify patients with more severe ARDS for their clinical trial of testing neuromuscular blockade [9]. Thus, whatever definitions are proposed, investigators who test new therapies need to decide which patients are most appropriate for the therapeutic intervention. One omission of the Berlin Definition is the failure to include patients with early acute lung injury who are not already receiving positive pressure ventilation, a group that is of growing importance in the field.

### Insights into genetic factors

Over the past eight years, there has been considerable work to test the contribution of genetic factors that might increase the risk of developing ALI/ARDS or be associated with worse outcomes. Most of the studies have been based on a candidate gene approach. An excellent review article described recent advances in genetic predisposition to clinical acute lung injury in 2009 [10]. Since this field emerged, variants in more than 30 genes have been associated with increased risk for developing ALI/ARDS, and also with worse clinical outcomes. The genetic factors have been single nucleotide polymorphisms that can regulate coagulation, inflammation, generation of reactive oxygen species, endothelial cell permeability, and apoptosis [11–13]. A recent study from our group indicated that African American patients with ALI have a higher risk for mortality compared with white patients. We used a candidate gene approach and identified a functional T-46C polymorphism (rs2814778) that is present in the promoter region of the Duffy antigen/receptor for chemokines (*DARC* gene) that was associated with an increase in 60-day mortality (17%) in African-American patients enrolled in the NHLBI ARDS Network clinical trials. Plasma interleukin-8 levels were also increased in those individuals with the *DARC* polymorphism [14]. Further work will be done to test the role of interleukin-8 and other pro-inflammatory cytokines in mediating this association, as well as testing the overall hypothesis that African-American patients have an excessive inflammatory response to the conditions that lead to ALI/ARDS, including sepsis and pneumonia.

Interesting work is also being done with the Fas pathway that modulates apoptosis and potentially lung epithelial cell injury. In one candidate gene study, genetic variants in Fas were associated with susceptibility to developing ALI [12]. Other studies have identified candidate genes for angiopoietin-2 that may play an important role in susceptibility or severity of lung injury [11]. Also, one study used a limited genome-wide association approach and identified a single-nucleotide polymorphism (*PPF1A1*), which encodes liprin-A. This protein is involved in cell-matrix interactions and integrin expression [15]. *PPF1A1* was a predictor for developing acute lung injury after major trauma. In addition, there is also a genome-wide association study in progress directed by Dr. Mark Wurfel at the University of Washington in Seattle, which is intended to identify genetic factors that may predispose certain patients to developing acute lung injury by studying patients with ALI as well as controls from multiple sites in the U.S.

### Environmental influences

The potential importance of environmental factors in the development of ALI/ARDS has been tested for two major factors: chronic alcohol abuse and more recently cigarette smoke exposure. Chronic alcohol abuse increases the risk of ALI and ARDS and also multiple organ failure and septic shock [16]. Recent work by Calfee et al. has demonstrated that both active and passive cigarette smoke exposure are independently associated with the development of ALI after severe blunt trauma [17]. The definition of active and passive cigarette smoke exposure in this study was reached by measuring plasma levels of cotinine, a major advance in being able to quantify passive or active cigarette smoke exposure [18]. The ability to quantify cigarette smoke exposure paves the way for further studies that can test the association of cigarette smoke exposure (either active or passive) in other patient populations at risk of developing ALI/ARDS or with acute lung injury, including sepsis, pneumonia, or aspiration. The mechanisms that explain why cigarette smoke exposure makes patients susceptible to developing lung injury will require further clinical research work, testing candidate biologic pathways of lung endothelial and alveolar epithelial injury, as well as biologic markers of acute inflammation and neutrophil and platelet function. It is clear that platelets can contribute with neutrophils to the development of acute lung injury, and since cigarette smoke can alter platelet function, there may be an important link in this regard [19].

### Physiologic predictors of outcome

Hypoxemia is recognized as the primary physiologic abnormality in patients with ARDS. Hypoxemia is caused by low ventilation to perfusion mismatching and right-to-left intrapulmonary shunting from airspaces that are collapsed or filled with edema fluid. In addition to hypoxemia, another important physiologic characteristic of ARDS is an impaired ability to excrete carbon dioxide. Approximately 10 years ago, our research group completed a prospective study of 179 patients with ARDS (PaO_2_/FiO_2_ < 200 mm Hg) to test the hypothesis that an early elevation in the pulmonary dead space fraction would be associated with increased mortality [20]. The measurements were made in patients within 24 hours of the diagnosis of ARDS. The results showed a significant increase in the pulmonary dead space fraction in those patients who ultimately died compared to those who survived. This association was confirmed by multivariate analysis and interestingly, the degree of arterial hypoxemia as measured by PaO_2_/FiO_2_ ratio was not associated with mortality. A decrease in static respiratory compliance was also independently associated with mortality. Subsequent work has confirmed that elevated pulmonary dead space is associated with mortality even in the era of lung-protective ventilation [21]. Thus, hypoxemia and impaired carbon dioxide excretion are characteristic physiologic features of ARDS. The decreased ability to excrete carbon dioxide is probably related to ventilation of lung units that have reduced blood flow, as well as lung units that are fluid-filled or collapsed.

In addition, single-center studies from our group as well as those from the NHLBI ARDS Network have indicated that oxygenation index ([mean airway pressure × FiO_2_ × 100] / PaO_2_) is an excellent predictor of outcome in patients with acute lung injury. In the ARDS Network Fluid and Catheter Treatment Trial (FACTT), the patients who were treated with a fluid conservative strategy had progressive improvement in their oxygenation index that seemed to reflect a likely decrease in pulmonary edema and a greater likelihood of being liberated from mechanical ventilation [22]. We carried out a study of 149 patients in two hospitals with ALI/ARDS and identified significant predictors of mortality in a bi-variate analysis, and then carried out a multivariate analysis to identify independent predictors of mortality. Overall, hospital mortality was 41%. In the multivariable-adjusted analysis, the PaO_2_/FiO_2_ ratio was not a statistically significant predictor of death, with an odds ratio (OR) of 1.29 (confidence interval, CI, 0.82-2.02). In contrast, the oxygenation index was a statistically significant predictor of death in both the unadjusted and the adjusted analyses (OR = 1.84, CI = 1.13-2.99) [23]. Thus, the oxygenation index may be a superior predictor because it integrates both airway pressure and oxygenation into a single variable. This study did confirm that demographic and laboratory variables identified in prior studies, including age, APACHE II, cirrhosis, and arterial pH, were also predictive of death.

### Pathogenesis

Several recent articles have focused on pathogenesis of injury to the pulmonary microcirculation and the alveolar epithelium [1, 24–26]. One area of particular interest is the potential role of integrins in modulating endothelial permeability in the lung. In an experimental study in mice, a blocking antibody against the integrin αvβ5 prevented an increase in lung vascular permeability in two models of ALI, ischemia-reperfusion in rats and in ventilation-induced lung injury in mice [27]. The knockout mice that were homozygous for a known mutation of the integrin β5 sub-unit were also protected from an increase in lung vascular permeability in a ventilator-induced lung injury model. *In vitro* studies with isolated pulmonary endothelial cells confirmed these findings. Actin stress fibrin formation induced *in vitro* by VEGF, TGF-β, and thrombin was reduced by blocking αvβ5 [27]. In contrast, αvβ3 appears to be an important integrin for maintaining normal lung vascular permeability. Blockade of the αvβ3 integrin prevents the barrier-protective effect of lipid sphingosine-1-phosphate (S1P) when added to thrombin-treated endothelial monolayers [28]. Further, integrin β3-null mice have an increased lung vascular permeability and mortality in endotoxin and sepsis models of lung injury. More recent work indicates that IQGAP1 may play an important role in regulating lung endothelial permeability as well [29]. Like integrin β3, IQGAP1 localizes to the endothelial cell junction after S1P treatment and IQGAP knockdown prevents corticoactin formation and barrier enhancement in response to S1P. Further, IQGAP-1-null mice develop increased lung vascular permeability to protein compared to wild-type controls over 72 hours after intratracheal instillation of endotoxin. In an E. coli pneumonia model, the IQGAP knockout mice had an increase in lung weight, lung water, and extravascular protein accumulation. There are several other mechanisms of lung injury that have been discussed in other review articles [1].

### Pathology

**Figure 1:**
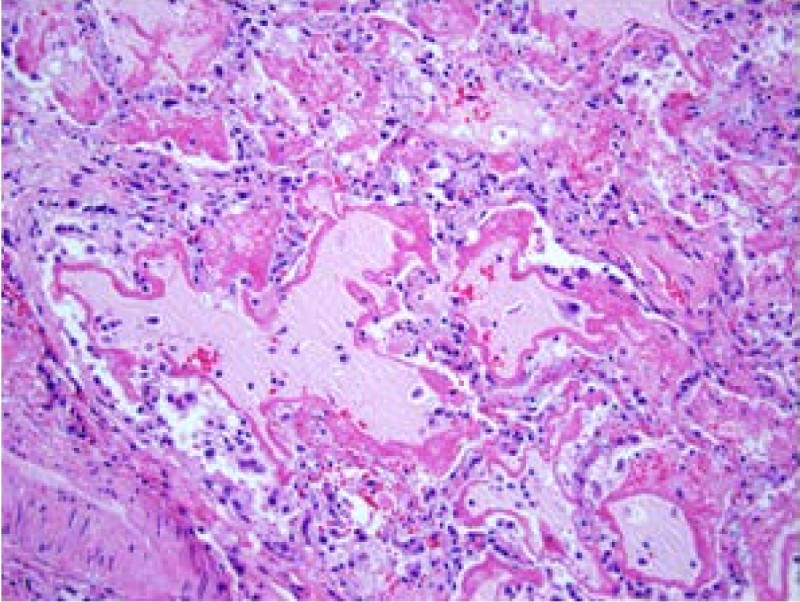
**Lung biopsy from a patient with ARDS from sepsis.** Note the pink proteinacious edema fluid filling the alveoli with classic deposition of hyaline membranes along the rims of the alveoli. There are also monocytes, macrophages, and neutrophils as well as probable denuded alveolar epithelial cells. These findings are consistent with the acute inflammatory and exudative phase of acute lung injury, pathologically termed diffuse alveolar damage. The section was stained with hematoxylin and eosin and the magnification is 200 x.

The classic pathologic findings in the lungs of patients who died from ARDS were described in 1977 by Bachofen and Weibel [30]. They divided the pathologic findings into the acute phase (the first six days), in which there was a predominance of both alveolar and interstitial neutrophilic infiltrate along with protein-rich edema and red blood cells. At this time, ultrastructural studies showed evidence of lung endothelial and alveolar epithelial injury. The alveolar epithelium was often completely denuded and hyaline membranes were present. See Figure 1 for an example of this acute phase. In the sub-acute phase (7–14 days), there was evidence of some edema reabsorption along with proliferation of alveolar epithelial type II cells in an effort to repopulate the injured epithelium. There was also evidence of fibroblast infiltration and some collagen deposition. There were also some patients who died after 14 days in what was termed the ‘chronic’ phase, in which the acute inflammation was resolved but there was a predominance of mononuclear cells, alveolar macrophages, fibroblasts, and some evidence of fibrosis. In the post-lung-protective ventilation era, there are fewer patients who die during the chronic phase, and there is less evidence of the fibrosing alveolitis than in the past, perhaps because the severity of patients’ lung injury is no longer markedly worsened by unfavorable high tidal volume and high airway pressure ventilation.

A recent study was published that evaluated 712 autopsies over a period of two decades, with a focus on evaluating the pathologic findings in relation to the recent proposed Berlin Definition of severity of ARDS [31]. The classic definition of ARDS histologically has depended on the presence of hyaline membranes. In this study, the authors found that only 45% of patients with a clinical diagnosis of ARDS had diffuse alveolar damage on pulmonary histology at autopsy. However, if the pathologic findings of pneumonia were included, then 88% of the ARDS cases as defined by the Berlin Definition had diffuse alveolar damage or histologic findings of pneumonia. Because pneumonia is the most common clinical cause of ARDS, the addition of criteria for pneumonia to the pathologic findings for ARDS appeared to be reasonable and important for future studies of ARDS. One of the major findings from this study was the observation that a lower proportion of diffuse alveolar damage was found in patients who underwent autopsies in the past decade (2001–2010) compared to those in the initial decade of 1991–2000 [32]. Patients in the more recent cohort in the past ten years were more likely ventilated with a lung-protective strategy in comparison to the patients who underwent autopsy from 1991 to 2000. Thus, the results indicate that lower tidal volume reduces the development of diffuse alveolar damage and more severe lung injury, which is consistent with considerable experimental and clinical work in ARDS.

Interestingly, patients who survive ARDS usually recover with near-normal lung function over the course of a year, but they often have cognitive impairment or muscle weakness [33].

### Treatment

Three randomized clinical trials have reported that lung-protective ventilation with reduced tidal volume and airway pressures decreased mortality in ALI/ARDS [34–36]. The decrease in mortality is quite remarkable when viewed over the course of the past 10 years, when lung-protective ventilation has become the standard of care in the treatment of most patients with ALI/ARDS in modern intensive care units. Mortality rates in the NHLBI ARDS Network have fallen from 40% to approximately 21-22% [1]. Other advances include better strategies to prevent nosocomial pneumonia, nosocomial catheter infections, and more selective use of blood products have contributed to this decline in mortality [37], but the major benefit is probably explained by the increasing use of lung-protective ventilation. In addition, a well done multi-center trial from France demonstrated that the use of neuro-muscular blockade in patients with more severe ARDS (PaO_2_/FiO_2_ < 150 mm Hg) further reduced mortality [9], probably because of greater success in instituting lung-protective ventilation, although other mechanisms have not been ruled out.

The additional therapeutic approach that has improved clinical outcomes in ALI/ARDS is the use of a fluid conservative strategy once shock has been resolved. Based on experimental studies, a reduction in lung vascular hydrostatic pressure decreases pulmonary edema in the setting of increased lung vascular permeability [38]. The NHLBI ARDS Network trial of 1,000 patients reported that a fluid conservative strategy significantly reduced the average duration of mechanical ventilation by 2.5 days [39].

Other phase II and III clinical trials did not show a treatment benefit from a wide variety of approaches, including surfactant treatment, inhaled nitric oxide, methylprednisolone, inhaled or intravenous beta-agonist therapy and activated protein C [1].

Several trials are currently in progress, including testing the potential therapeutic value of statin therapy for ALI/ARDS, because of its demonstrated ability to decrease inflammation and restore vascular permeability toward normal. In the future, trials are planned with allogeneic human mesenchymal stem cells for severe ARDS and advances in extracorporeal membrane oxygenation therapy (ECMO) have made it possible to rescue some patients with very severe ARDS who have primarily single-organ failure [40].

## Review and conclusions

Progress has been made in identifying patients with acute lung injury prior to their admission to the intensive care unit and prior to the institution of positive pressure ventilation, facilitating testing of new therapies at an early stage of the clinical disorder. The Berlin Definition represents progress in simplifying the diagnosis of ARDS into logical categories by the severity of arterial hypoxemia. The identification of cigarette smoke exposure plus alcohol use as important environmental factors that increase susceptibility to developing ARDS represents solid progress in understanding the risk factors for developing ARDS. Genetic factors may increase the risk of developing ARDS or its severity, but only limited progress has been made to date, in part because of the heterogeneity of the ARDS phenotype. Several large studies have identified some pulmonary specific variables that have some predictive value for severity of lung injury and clinical outcomes, including elevated pulmonary dead space and also elevated oxygenation index. The clinical application of lung protective ventilation transformed the treatment of ARDS, leading to a marked decrease in mortality, with complementary evidence that the severity of lung injury histologically is less. New therapeutic strategies are being evaluated currently in several phase 2 and 3 trials.

## Authors’ original submitted files for images

Below are the links to the authors’ original submitted files for images. Authors’ original file for figure 1
