# Supplementary figures and images for: The acute respiratory distress syndrome in 2013
Source: Transl Respir Med. 2013 May 17;1:10. doi: 10.1186/2213-0802-1-10 (PMC6733424; doi:10.1186/2213-0802-1-10)

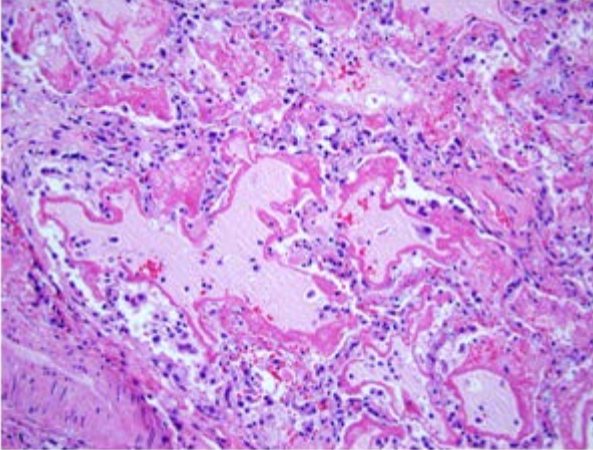

Supplement: Supplementary file 1 — Authors’ original file for figure 1 [file 40247_2013_10_MOESM1_ESM.pdf]
